# Effects of multiple genetic loci on the pathogenesis from serum urate to gout

**DOI:** 10.1038/srep43614

**Published:** 2017-03-02

**Authors:** Zheng Dong, Jingru Zhou, Shuai Jiang, Yuan Li, Dongbao Zhao, Chengde Yang, Yanyun Ma, Yi Wang, Hongjun He, Hengdong Ji, Yajun Yang, Xiaofeng Wang, Xia Xu, Yafei Pang, Hejian Zou, Li Jin, Jiucun Wang

**Affiliations:** 1 State Key Laboratory of Genetic Engineering, Collaborative Innovation Center for Genetics and Development, School of Life Sciences, Fudan University Jiangwan Campus, Shanghai, China; 2 Division of Rheumatology and Immunology, Changhai Hospital, Shanghai, China; 3 Division of Rheumatology, Ruijin Hospital, Shanghai Jiaotong University School of Medicine, Shanghai, China; 4 Division of Rheumatology, Taixing People’s Hospital, Jiangsu Province, China; 5 Division of Rheumatology, Taizhou People’s Hospital, Jiangsu Province, China; 6 Fudan-Taizhou Institute of Health Sciences, Taizhou, Jiangsu Province, China; 7 Division of Rheumatology, Huashan Hospital, Fudan University, Shanghai, China; 8 Institute of Rheumatology, Immunology and Allergy, Fudan University, Shanghai, China

## Abstract

Gout is a common arthritis resulting from increased serum urate, and many loci have been identified that are associated with serum urate and gout. However, their influence on the progression from elevated serum urate levels to gout is unclear. This study aims to explore systematically the effects of genetic variants on the pathogenesis in approximately 5,000 Chinese individuals. Six genes (*PDZK1, GCKR, TRIM46, HNF4G, SLC17A1, LRRC16A*) were determined to be associated with serum urate (*P*_FDR_ < 0.05) in the Chinese population for the first time. *ABCG2* and a novel gene, *SLC17A4*, contributed to the development of gout from hyperuricemia (OR = 1.56, *P*_FDR_ = 3.68E-09; OR = 1.27, *P*_FDR_ = 0.013, respectively). Also, *HNF4G* is a novel gene associated with susceptibility to gout (OR = 1.28, *P*_FDR_ = 1.08E-03). In addition, *A1CF* and *TRIM46* were identified as associated with gout in the Chinese population for the first time (*P*_FDR_ < 0.05). The present study systematically determined genetic effects on the progression from elevated serum urate to gout and suggests that urate-associated genes functioning as urate transporters may play a specific role in the pathogenesis of gout. Furthermore, two novel gout-associated genes (*HNF4G* and *SLC17A4*) were identified.

Gout is among the most common forms of inflammatory arthritis and affects approximately 1 to 6% of the population in various countries^1^,^2^,^3^. An elevated serum uric acid concentration (hyperuricemia, HUA) promotes the deposition of monosodium urate crystals in the joints, which then causes gout^4^,^5^. Hyperuricemia is a key risk factor for the pathogenesis of gout^6^, but only a quarter of people with hyperuricemia develop gout, suggesting that an elevated serum uric acid concentration is necessary but not sufficient for the pathogenesis of gout^7^,^8^. Recently, a large meta-analysis of genome-wide association studies (GWAS) identified 28 loci associated with serum urate concentration^9^; however, this result only explained approximately 7% of the variance in serum urate concentrations, and only a portion of those loci were determined to be associated with the risk of gout^9^,^10^. Therefore, it is necessary to systematically analyze genetic effects on the progression from elevated serum urate to gout and to further identify novel candidate loci that affect the risk of HUA and gout. In addition, population groups have been reported to show common heterogeneity in the genetic contribution of serum urate concentrations and gout^1^,^11^,^12^, suggesting the need for transancestral studies to identify population-specific loci that affect the pathogenesis of gout.

To explore the genetic architecture of serum urate and gout mentioned above, 31 SNPs were selected based on predefined criteria and tested in 4914 Chinese individuals (582 gout patients, 1387 HUA patients and 2945 healthy subjects). For data analysis, genetic effects on different combinations (serum urate, gout vs. control, gout vs. HUA and gouty tophi vs. control) were tested to show the influence of genetic variants on the progression from elevated serum urate to gout. In addition, the differences in the transcription levels of candidate genes were measured in 213 male individuals (70 gout patients, 85 hyperuricemia patients and 58 healthy subjects) to further confirm the above results. Finally, this study systematically analyzed the genetic effects on the pathogenesis of gout from elevated serum urate.

## Results

### Loci associated with serum urate and gout

The process for selecting target loci is shown in Figure S1, and after filtration, 31 loci were tested in 4914 Chinese individuals (582 gout patients, 1387 HUA patients and 2945 healthy subjects).

Thirteen loci in ten genes (*TCF7L2, A1CF, PDZK1, GCKR, ABCG2, SLC2A9, TRIM46, HNF4G, SLC17A1* and *ESR1*) were determined to be associated with serum urate, with *P* values below 0.05 (Table 1). Among them, eleven loci in eight genes (*TCF7L2, PDZK1, GCKR, ABCG2, SLC2A9, TRIM46, HNF4G*, and *SLC17A1*) were still associated with serum urate after multiple correction (all *P*_FDR_ < 0.05). To the best of our knowledge, six genes (*PDZK1, GCKR, TRIM46, HNF4G* and *SLC17A1*) were identified in the Chinese population for the first time.

In addition, twelve loci in nine genes (*A1CF, PDZK1, GCKR, ABCG2, SLC2A9, TRIM46, HNF4G, SLC17A1* and *SLC17A4*) were found to influence the risk of gout (all *P* < 0.05) (Table 1). After multiple correction, except for the genes *PDZK1* and *SLC17A4*, the remaining ten genes still had effects on the risk of gout with a *P*_FDR_ value less than 0.05. *HNF4G* is a novel gout-associated gene associated with the pathogenesis of gout (OR = 1.28, *P*_FDR_ = 1.08E-03), and two other genes, *A1CF* and *TRIM46*, were identified to be associated with susceptibility to gout in the Chinese population for the first time (rs10821905 in *A1CF*: OR = 1.61, *P*_FDR_ = 1.57E-03; rs4971101 in *TRIM46*: OR = 1.37, *P*_FDR_ = 3.25E-04; rs2070803 in *TRIM46*: OR = 1.22, *P*_FDR_ = 0.031). Because *SLC17A4* did not affect the concentration of serum urate (*P*_FDR_ = 0.108), the combined sample of HUA patients and healthy controls was treated as a larger sample control for the further analysis of gout. As a result, *SLC17A4* was found to be a novel gout-associated gene affecting the risk of gout (OR = 1.19, *P*_FDR_ = 0.018). For gouty tophi case compared with controls, six genes (*A1CF, NRXN2, GCKR, ABCG2, SLC17A1* and *TRIM46*) were found to influence the development of gouty tophi (all *P* < 0.05), and after multiple correction, three genes (*A1CF, ABCG2* and *TRIM46*) were still associated with gouty tophi (all *P*_FDR_ < 0.05) (Supplementary Material, Table S1). In addition, to avoid the heterogeneity of gender, logistic regression adjusted for gender was performed to confirm the association for *HNF4G* and *SLC17A4*, and the results also showed that those genes influence the risk of gout (*P*_FDR_ = 8.92E-05 and 0.040, respectively).

To further understand the pathogenesis of gout from serum urate, the comparative genetic effects in hyperuricemia patients and gout patients were analyzed. Six genes (*A1CF, ABCG2, SLC17A1, TRIM46, ADRB3* and *SLC17A4*) were found to be associated with this pathogenesis (all *P* < 0.05) (Table 1). After multiple correction, the association between two genes, *ABCG2* and *SLC17A4* (novel gout-associated gene), remained significant (OR = 1.56, *P*_FDR_ = 3.68E-09; OR = 1.27, *P*_FDR_ = 0.013, respectively).

### Association between genetic variants and serum urate or gout in gender subgroups

Gender has been proven to be an important heterogeneity factor for serum urate and gout^1^,^9^. Thus, we further tested the above associations in subgroups of gender (Table 2). In the male subgroup, nine loci in six genes (*TCF7L2, GCKR, ABCG2, TRIM46, HNF4G* and *SLC17A1*) were determined to be associated with serum urate (all *P* < 0.05), and four genes (*GCKR, ABCG2, HNF4G* and *SLC17A1*) were still associated with serum urate after multiple correction (all *P*_FDR_ < 0.05). Eight loci in six genes (*TCF7L2, ABCG2, SLC2A9, TRIM46, ESR1* and *SLC17A4*) exhibited contributions to the level of serum urate in females, with a *P* value less than 0.05, and *ABCG2* showed significant association after multiple corrections (rs1481012: *P*_FDR_ = 7.47E-10).

Six genes (*A1CF, ABCG2, SLC2A9, TRIM46, SLC17A1* and *SLC17A4*) exhibited effects on the development of gout from hyperuricemia in males (all *P* < 0.05), but none of them exhibited such effects in females (all *P* > 0.05) (Table 2). After multiple correction, only the association of *ABCG2* in males was still significant (OR = 1.69, *P*_FDR_ = 1.55E-10).

In the male subgroup, nine genes (*A1CF, PDZK1, GCKR, ABCG2, SLC2A9, TRIM46, HNF4G, SLC17A1* and *SLC17A4*) were determined to be associated with gout risk (all *P* < 0.05) (Table 2), which was consistent with the results of the association in all samples (Table 1). Six of the genes (*A1CF, GCKR, ABCG2, TRIM46, SLC17A1* and *HNF4G* (novel gout-associated gene)) were still significantly associated with gout in males after multiple correction (all *P*_FDR_ < 0.05). Because several loci did not affect the concentration of serum urate in males, the combined sample of hyperuricemia patients and healthy controls was treated as a larger sample control for further analysis. Consequently, another novel gout-associated gene, *SLC17A4*, was identified as a risk factor for the pathogenesis of gout in males (OR = 1.19, *P*_FDR_ = 0.035) (Table 2). In females, *ABCG2* and *TRIM46* contributed to the risk of gout (both *P* < 0.05), but neither was significant after multiple correction (both *P*_FDR_ > 0.05).

### Association between genetic variants and serum urate in BMI and smoking subgroups

Previous studies have shown that obesity and cigarette smoking can influence serum uric acid levels^13^,^14^,^15^, while their effect on the association between genetic variants and urate was limited. Therefore, further analysis in the BMI and smoking status subgroups was performed.

When body mass index (BMI) was analyzed, ten loci in seven genes (*TCF7L2, GCKR, ABCG2, SLC2A9, TRIM46, SLC17A1* and *LRRC16A*) were associated with serum urate in normal individuals (18.5 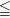 BMI < 25), with a *P*_FDR_ value less than 0.05, and three of those genes (*GCKR, ABCG2* and *TRIM46*) were associated with serum urate in overweight subjects (BMI ≥ 25) (Table 3). To the best of our knowledge, *LRRC16A* was identified in the Chinese population for the first time. In the underweight subgroup, no significant associations were found after multiple correction.

*ABCG2* affected the serum uric acid level in all subgroups of smoking status (non-smokers, former smokers and current smokers), suggesting its strong role in influencing of serum urate concentrations (rs1481012: beta = 16.925, *P*_FDR_ = 0.027; beta = 15.645, *P*_FDR_ = 5.76E-04; beta = 26.700, *P*_FDR_ = 1.94E-15, respectively) (Table 3). *HNF4G* was associated with serum urate concentration in individuals who were former smokers (beta = 11.394, *P*_FDR_ = 0.020) but not in the other subgroups non-smokers: beta = 1.411, *P*_FDR_ = 0.944; smokers: beta = 2.385, *P*_FDR_ = 0.855). In addition, *GCKR* and *TRIM46* only modified the serum urate level in smoking subjects (rs1260326: beta = 9.632, *P*_FDR_ = 2.88E-03; rs2070803: beta = 7.541, *P*_FDR_ = 0.040, respectively).

### The contribution of genetic effects to the pathogenesis of gout

**Figure 1:**
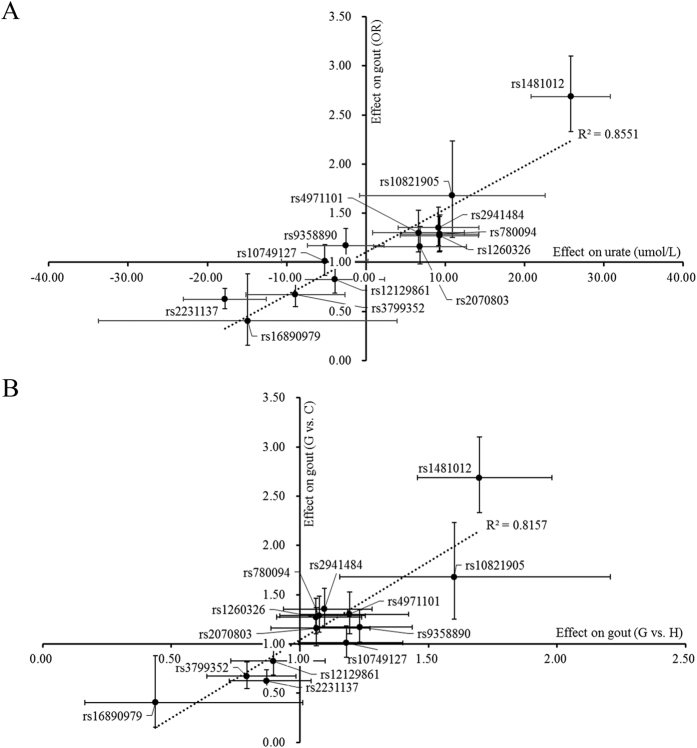
Relationship between genetic effects on serum urate and gout across all 13 loci in males. (**A**) urate beta coefficients and gout odds ratios; (**B**) odds ratios for gout vs. hyperuricemia and gout odds ratios. Each confidence interval for a beta coefficient or odds ratio estimate was plotted as a bar of the point.

Because most candidate genes only affected the concentrations of serum urate and the risk of gout in males (Table 2), further analysis for the candidate loci were only preformed in males. Across the 13 loci (rs10749127, rs10821905, rs12129861, rs1260326, rs1481012, rs16890979, rs2070803, rs2231137, rs2941484, rs3799352, rs4971101, rs780094 and rs9358890) identified above, for each additional effect allele in males, the odds ratio for gout showed positive linear correlation with the genetic effect on serum urate (R^2^ = 0.855) (Fig. 1A). This result was consistent with the fact that an increased serum uric acid level is a key risk factor in the pathogenesis of gout^9^. For the genetic contribution of hyperuricemia to gout, we tested the correlation between the genetic effects associated with gout and hyperuricemia/control. The results showed a high correlation between those two associations (R^2^ = 0.816), suggesting that the development of gout from hyperuricemia made a great contribution to the pathogenesis of gout (Fig. 1B).

### Genetic urate risk score associated with hyperuricemia and gout

In males, the genetic urate score for the 13 loci identified above could explain an average of 4.76% of the serum urate variance and was strongly associated with hyperuricemia and gout (coefficients = 0.013, *P* < 2E-16; coefficients = 0.025, *P* < 2E-16, respectively). The scores ranged from −70 to + 140, and the increased genetic urate score resulted in elevated proportions of hyperuricemia and gout in males (Supplementary Material, Figure S2). Furthermore, the male subjects with scores higher than 80 showed a 16.44 (95% CI: 8.85–32.40) times higher risk for gout and 3.53 (95% CI: 2.35–5.35) times higher risk for hyperuricemia than male subjects with scores less than −10.

### Differential expression of candidate genes among groups

**Figure 2:**
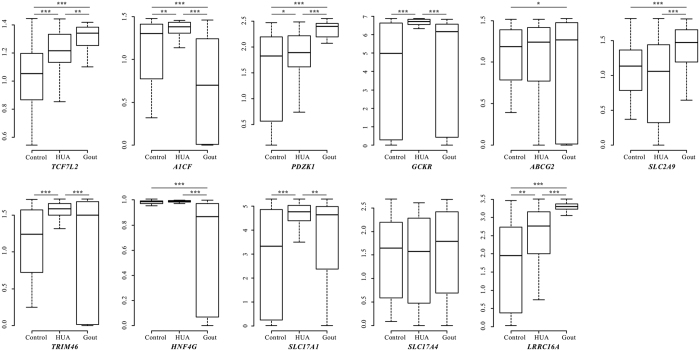
Differential expression of candidate genes among groups. SYBR Green-based quantitative polymerase chain reaction (qPCR) was used to test the relative mRNA levels of candidate genes. The mRNA expression data were analyzed by Student’s *t*-test. Data are illustrated as box plots. The upper and lower edges of each box represent the 75th and 25th percentiles, respectively. The lines inside the boxes represent the median.

To further validate the results presented above, this study randomly selected 70 male gout patients, 85 male hyperuricemia patients and 58 healthy male individuals and tested the differences in the transcription levels of the candidate genes among them (Fig. 2). All candidate genes, except for *SLC17A4*, showed at least one significant difference in relative expression with a *P* value less than 0.05, indicating that the loci identified above might influence the risk of hyperuricemia and gout through changing the relative expression levels. Regarding *SLC17A4*, the synonymous mutation of rs9358890 might affect mRNA transport, splicing and translation^16^ and thereby influence the pathogenesis of gout. In addition, the candidate loci (rs10821905, rs12129861, rs1260326, rs2070803, rs2231137, rs3799352, rs4971101, rs780094 and rs742132) were significantly expressed quantitative trait loci (eQTL) that were associated with the expression of one or more transcript in one or more tissue by querying two existing expression eQTL databases (Supplementary Material, Table S2).

### Genetic variants influence the progression from hyperuricemia to gout

**Figure 3:**
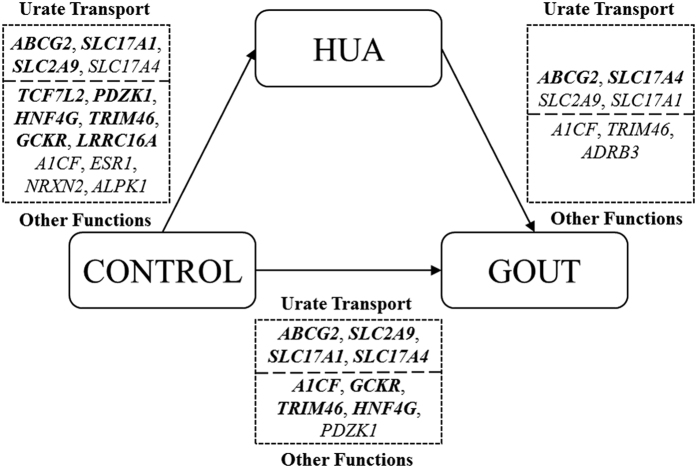
Systematic analysis of genetic variants influencing the progression from hyperuricemia to gout. HUA, hyperuricemia. All genes identified above with nominal significance were used in this analysis. Each gene was associated with a special phenotype with a *P*_FDR_ value less than 0.05, as shown in bold. *ABCG2, SLC2A9, SLC17A1* and *SLC17A4* could encode urate transport.

Across all of the genes identified above with nominal significance, a systematic analysis was conducted to determine the influence of genetic variants on the progression from elevated serum urate to gout (Fig. 3). As a result, a total of fourteen genes exhibited effects on the serum urate concentrations, seven genes on the development of gout from hyperuricemia, and nine genes on gout in all individuals or subgroups. Among the fourteen genes, six genes (*ABCG2, SLC2A9, TRIM46, SLC17A1, A1CF* and *SLC17A4*) affected both the elevation of the serum urate level and the development of gout from hyperuricemia and were identified as risk factors for gout. Interestingly, three genes (*PDZK1, GCKR* and *HNF4G*) associated with urate influenced the risk of gout, but the other five genes (*TCF7L2, LRRC16A, ESR1, NRXN2* and *ALPK1*) did not, suggesting that elevated serum urate concentration is necessary but not sufficient for the pathogenesis of gout (Fig. 3). In summary, different genes played distinct roles in the pathogenesis of gout, and the loci associated with both serum urate and the development of gout from hyperuricemia were definitively identified as risk factors for gout.

## Discussion

An elevated serum urate concentration is necessary but not sufficient for the pathogenesis of gout^7^,^8^. A total of 28 serum urate concentration-associated loci only explained approximately 7% of the variance in serum urate concentrations, and a portion of those loci were determined to contribute to the pathogenesis of gout^9^,^10^. Therefore, it is necessary to systematically analyze the genetic variants influencing the progression from elevated serum urate to gout and to identify novel candidate loci that affect the risk of gout. In addition, population-specific effects of serum urate concentrations and gout related genes have been proven before^1^,^11^, suggesting the need for transancestral studies in validating those loci in other population without association tests.

In this study, we systematically analyzed the genetic variants influencing the progression from elevated serum urate to gout and identified 2 novel gout-associated genes (*HNF4G* and *SLC17A4*), using genetic analysis and mRNA expression analysis. The loci from the association analysis were characterized in detail, including an analysis of the association between subgroups comprising gender, body mass index (BMI) and smoking status. For each effect allele identified in this study, the genetic effect on serum urate showed a positive linear correlation with the odds ratio for gout. Gene expression analysis further validated the associations for the loci identified above (except for *SLC17A4*).

Ten genes previously reported to be associated with serum urate and/or gout^8^,^9^,^10^,^17^,^18^,^19^, were confirmed in this study and further analyzed by mRNA expression analysis. To the best of our knowledge, of these genes, six urate concentration-associated genes (*PDZK1, GCKR, TRIM46, HNF4G, SLC17A1* and *LRRC16A*) and two gout-associated genes (*A1CF* and *TRIM46*) were identified in the Chinese population for the first time. In addition, two novel gout-associated genes, *HNF4G* and *SLC17A4*, were found to affect the risk of gout in our study.

*HNF4G* was reported as a urate-concentration gene and showed no evidence of association with gout in previous studies^9^,^10^, although a strong trend towards the association of *HNF4G* with gout had been found in Europeans (*P* = 0.058)^10^. HNF4G is a transcription factor responding to nutrient signals, and its overexpression in bladder tumors can significantly increase tumor cell viability, colony formation rate, and invasion, while HNF4G knockdown can achieve the reverse effects^20^. In addition, HNF4G can constitutively bind to endogenous fatty acids^21^. Our study shows that *HNF4G* gene expression to be lower in gout patients than in healthy individuals, most likely explaining the mechanism of its effect on the pathogenesis of gout. The effect mechanism should be further studied in future work.

SLC17A4 (NPT homologue), an intestinal organic anion exporter, belongs to the NPT subfamily, and its mRNA is expressed mainly in the pancreas, liver, colon, and intestine^22^,^23^. Togawa *et al*. found that SLC17A4 actually exists in the apical membrane of the small intestine and transports various types of organic anions, such as urate^22^. Urate is synthesized predominantly in the liver, and nearly two-thirds of daily urate excretion occurs via the kidneys^24^. The remaining urate may be excreted from the intestines, resulting from the biological function of urate excretion for SLC17A4 and BCRP, which are expressed in the intestines^22^,^25^. A meta-analysis of 28,141 individuals identified an additional larger region including the *SLC17A1, SLC17A3* and *SLC17A4* genes that influences the serum urate level^18^. In this study, *SLC17A4* was found to be associated with both gout and the development of gout from hyperuricemia, which partially explains the mechanism of the progression from hyperuricemia to gout.

Environmental factors, including gender, BMI and smoking status, commonly act as heterogeneity factors for the association of genetic variants and serum urate/gout^6^,^14^,^15^,^26^,^27^. For example, our previous study showed gender was a source of heterogeneity for the association between *ABCG2* variant and gout risk in both meta-regression and subgroup analyses, and the OR values in men and women were significantly different^1^. In this study, *HNF4G, SLC17A1* and *GCKR* played an important role in serum urate concentrations and gout risk in males but not in females, also suggesting the different contributions of genetic effects between different genders. Heterogeneity analysis was shown to determine the potential reasons for the equivocal results of the associations seen in our previous study^6^. For instance, a meta-analysis of genome-wide association studies suggested an association between uric acid and rs742132, a common variant in *LRRC16A*^18^; in contrast, a replication study with 7795 subjects showed no significant association between this locus and serum urate concentrations^28^. In our study, we also found that rs742132 exhibited no significant effect on serum urate. However, when analyzing by BMI subgroup, this locus showed a significant influence on serum urate concentrations in individuals with normal weight, indicating that the association between rs742132 and serum urate might be modified by BMI, and BMI might thus be a heterogeneity factor leading to the observed discrepancies in results. Above all, the subgroup analysis of heterogeneity factors was helpful to find associations concealed in complex data and to partially explain the biological mechanism of gout incidence via the interaction of genetic variants and environment factors.

One aim of this study was to systematically analyze genetic effects on the progression from hyperuricemia to gout. All the urate transporter-coding genes, *ABCG2, SLC2A9, SLC17A1* and *SLC17A4*, showed association with both the serum urate concentration and progression from hyperuricemia to gout and could affect the risk of gout (Fig. 3). However, only some of the other urate associated genes, which did not code urate transporters, were found to influence the development of gout. Thus, the difference in biological functions of urate-associated genes might be part of the reason that only a quarter of individuals with hyperuricemia can develop gout. In addition, we speculated that the urate-associated genes that function as urate transporters played a certain role in the pathogenesis of gout.

However, there were several limitations in this study. First, this study focused on only common SNPs and did not consider the contributions of rare variants, such as *ALDH16A1*^29^. Second, because SNPs that did not satisfy the requirements for selection criteria were replaced by other SNPs in the same genes or deleted directly, some SNPs enrolled in this study were not identical to the SNPs published in previous studies. In addition, the female sample is limited as the lower frequency of gout in females, the study might not have enough power to detect significant associations in females. Finally, although gender, BMI and smoking status were considered in our analysis, other environmental factors associated with uric acid and gout were not assessed. Therefore, further studies of these loci should be performed.

In conclusion, this study systematically revealed the genetic effects on the pathogenesis of gout from elevated serum urate and identified two novel gout-associated genes (*HNF4G* and *SLC17A4*). The loci associated with increased levels of uric acid were also associated with an increased risk of gout. This study suggests that the differences in biological function of urate-associated genes might be the reason that only a quarter of individuals with hyperuricemia develop gout. We also speculate that the urate-associated genes that function as urate transporters played a certain role in the pathogenesis of gout. These findings strongly support the hypothesis that genetic variants in urate transport genes are the key factors affecting the concentration of serum urate and the risk of gout, suggesting potential implications for the prevention, prediction and treatment of hyperuricemia and gout.

## Materials and Methods

### Experimental Design

Although genome-wide association studies have identified many genes which play a role in serum urate and gout, our understanding of the effect of genetic variants on the pathogenesis of hyperuricemia and gout is quite limited. Here, we systematically analyzed the genetic variants influencing the progression from elevated serum urate to gout using genetic analysis and mRNA expression analysis. By examining approximately 5,000 Chinese individuals, we attempted to identify novel gout-associated genes and systematically analyzed the genetic effects on the pathogenesis of gout. Further analysis for the transcription levels of the candidate genes also was used to study the association of candidate genes with gout. In addition, we also analyzed the effect of heterogeneity factors (gender, body mass index and smoking status) on the association between genetic variants and serum urate concentrations.

### Study subjects

This study was approved by the Ethical Committees of the School of Life Sciences of Fudan University and was conducted in accordance with the guidelines and regulations of the Declaration of Helsinki. All participants provided written informed consent to this study. A total of 582 gout patients and their clinical information were collected from Changhai Hospital, Taixing People’s Hospital and Taizhou People’s Hospital. All gout patients enrolled in this study were clinically diagnosed with primary gout (OMIM: #138900) according to the American College of Rheumatology diagnostic criteria^30^. All patients did not have urate-lowering drugs two weeks before sample collection. Among these patients, 174 gout patients were recorded with gouty tophi, which are deposits of uric acid crystals and pathognomonic for the disease gout.

In addition, 4332 subjects with no history of gout were recruited from the Taizhou Longitudinal Study^31^. Those individuals were divided into subgroups according to smoking status, as recorded in questionnaires, and body mass index (BMI) values following the categories of the World Health Organization (WHO)^32^. Smoking status included non-smokers, former smokers and current smokers. For BMI, three subgroups (underweight: BMI < 18.5; normal weight: 18.50 ≤ BMI < 25; overweight: BMI ≥ 25) contained 99, 2024 and 1825 individuals were used in this study. Among them, 1387 subjects with high serum urate (>417 umol/L) were treated as hyperuricemia patients^33^, and the rest of the patients were treated as healthy controls. The characteristics of the participants in this study are shown in Supplementary Material, Table S3 and Table S4.

### DNA extraction

Peripheral blood was collected from all participants enrolled in this study. Genomic DNA was isolated from whole blood using a QIAamp DNA Blood Mini kit (QIAGEN, Germany) and then stored at −20 °C immediately. The concentration and quality of DNA (including optical density (OD) 260/280 and 260/230 measurements) was determined using a Nanodrop Lite spectrophotometer (Thermo Fisher’s Scientific, Waltham, MA, USA).

### RNA Isolation, cDNA Synthesis, and Real-time qPCR

We randomly collected RNA from 70 male gout patients, 85 male hyperuricemia patients and 58 male healthy individuals. RNA was extracted from blood cells using TRIzol reagent according to the manufacturer’s instructions (Invitrogen, Carlsbad, CA, USA). Complementary DNA (cDNA) was synthesized through RNA reverse transcription using a High Capacity cDNA Reverse Transcription Kit (Applied Biosystems, Foster City, CA, USA) according to the manufacturer’s protocol. Real-time quantitative polymerase chain reaction (qPCR) was performed using SYBR Premix Ex Taq (TakaRa Biotech, Tokyo, Japan) with an ABI Prism 7900 Detector System (Applied Biosystems). The data obtained from the assays were analyzed using the SDS 2.3 software (Applied Biosystems). The human housekeeping gene glyceraldehyde-3-phosphate dehydrogenase (*GAPDH*) was used as an internal control.

### Target loci selected

The process for selecting target SNPs was as follows (Supplementary Material, Figure S1). First, SNP association studies were downloaded from the PubMed database (http://www.ncbi.nlm.nih.gov/pubmed/). Second, text-mining technology was used to search for SNPs associated with serum urate levels and/or gout and recorded the frequencies of reported associations for each SNP (frequency 1). Third, the same text-mining method was used to calculate the frequencies of associations with phenotypes other than serum urate levels and gout for each of the above SNPs (frequency 2). Fourth, each of the selected SNPs was considered as a candidate if its frequency 1 was significantly different with its frequency 2 with a *P* value less than 0.05 in Chi-squared test. In addition, those candidate SNPs were manually verified. Other reported urate/gout-associated SNPs in published reviews were also enrolled in our study. In addition, other important candidate SNPs in transporter genes and hypertension- or diabetes-related genes were included. All selected SNPs were annotated by SNPnexus (http://www.snp-nexus.org/) and filtered by their SNP functions (i.e. SNPs in 5′-upstream, 5′-utr, coding, intronic, 3′-utr and 3′-downstream were selected). Then, the SNPs were evaluated by the requirements of SNPscan, the genotyping technology used in our study. SNP that did not satisfy the requirements were replaced by other SNP in the same gene or deleted directly. Finally, after filtration, 31 SNPs were treated as target SNPs for further analysis.

### Genetic analysis

Peripheral blood was collected from all the individuals investigated in this study. Genomic DNA was isolated from whole blood using the QIAamp DNA Blood Mini kit (QIAGEN, Germany) and stored at −20 °C. The DNA concentration and quality (including optical density (OD) 260/280 and 260/230 measurements) were determined using a Nanodrop Lite spectrophotometer (Thermo Fisher Scientific, Waltham, MA, USA). Genotyping of all target SNPs was performed using SNPscan (TianHao, China).

### Genetic urate risk score analysis

To analyze the cumulative effects of the loci identified from the association studies for urate and gout, we multiplied, for each locus, the number of effect alleles each person carried (0–2) by the beta coefficient from the genetic analysis and added the results to obtain a genetic urate score^19^. The genetic urate score equation is as follows: rs10749127(N) × beta + rs10821905(N) × beta + rs12129861(N) × beta + rs1260326(N) × beta + rs1481012(N) × beta + rs16890979(N) × beta + rs2070803(N) × beta + rs2231137(N) × beta + rs2941484(N) × beta + rs3799352(N) × beta + rs4971101(N) × beta + rs780094(N) × beta + rs9358890(N) × beta, where N of each SNP denotes the number of copies of that allele carried by each subject, and beta value is the effect size per allele in serum urate. The association between genetic urate score and HUA or gout were tested in males by logistic regression with adjustment for age. Linear regression was used to analyze the relationship between the score and serum urate concentration in males.

### Statistical analysis

The genotype data of the loci were checked for deviation from the Hardy-Weinberg equilibrium. The beta values for serum urate loci were calculated by linear regression adjusted for age and gender. *P* values for serum urate loci were calculated by deviance analysis for linear regression with adjustment for age and gender. All *P* values for gout loci were calculated by Fisher’s exact test in the addition model and logistic regression adjusted for gender. Furthermore, subgroups based on gender, BMI and smoking status were used in this study. *P* values for the loci were multiply corrected by the FDR method (*P*_FDR_), and values below 0.05 were considered statistically significant.

Data on mRNA expression are illustrated as boxplots with all outliers cleared. The differences in mRNA expression of candidate genes among gout patients, hyperuricemia patients and healthy controls were analyzed by Student’s *t*-test. The differences among different genotypes were also tested in this study. *P* values below 0.05 were considered statistically significant. In addition, we also queried two existing expression quantitative trait locus (eQTL) databases (Geuvadis data browser (http://www.ebi.ac.uk/Tools/geuvadis-das/)^34^ and Genotype-Tissue Expression Data Portal (http://www.gtexportal.org/home/))^35^ to analyze the association of candidate loci with transcript expression.

All statistical analyses were performed using R (Version 3.0.2: www.r-project.org/).

## Additional Information

**How to cite this article**: Dong, Z. *et al*. Effects of multiple genetic loci on the pathogenesis from serum urate to gout. *Sci. Rep.* **7**, 43614; doi: 10.1038/srep43614 (2017).

**Publisher's note:** Springer Nature remains neutral with regard to jurisdictional claims in published maps and institutional affiliations.

## Supplementary Material

Supplementary Information

## Figures and Tables

**Table 1:** Association between genetic variants and serum urate/gout.

SNP	Chr.	Position	Type	Gene	A1	A2	Serum Urate			Gout vs. HUA			Gout vs. Control		
							Β	*P*	*P*_FDR_	OR	*P*	*P*_FDR_	OR	*P*	*P*_FDR_
rs5438	1	9129620	5′ UTR	*SLC2A5*	T	C	−0.495	0.652	0.765	1.15	0.258	0.56	1.1	0.423	0.722
rs1061622	1	12252955	Coding	*TNFRSF1B*	G	T	−1.725	0.576	0.731	1.01	0.895	0.927	1.04	0.625	0.906
rs12129861	1	145725689	5upstream	*PDZK1*	A	G	−5.856	0.017	**0.045**	0.9	0.258	0.56	0.84	0.046	0.111
rs4971101	1	155157635	3downstream	*TRIM46*	G	A	7.667	1.49E-03	**7.18E-03**	1.2	0.024	0.139	1.37	3.36E-05	**3.25E-04**
rs2070803	1	155157715	3downstream	*TRIM46*	A	G	8.05	1.01E-03	**5.86E-03**	1.06	0.482	0.777	1.22	0.011	**0.031**
rs2027432	1	247578441	5upstream	*NLRP3*	A	G	−4.201	0.27	0.46	1.11	0.453	0.777	1.01	0.952	0.985
rs7512998	1	247583221	Intronic	*NLRP3*	C	T	−1.12	0.66	0.765	1.1	0.465	0.777	1.01	0.905	0.985
rs1260326	2	27730940	Coding	*GCKR*	T	C	8.304	1.34E-04	**1.30E-03**	1.08	0.288	0.56	1.27	3.44E-04	**1.43E-03**
rs780094	2	27741237	Intronic	*GCKR*	T	C	8.128	2.03E-04	**1.48E-03**	1.08	0.289	0.56	1.27	2.44E-04	**1.18E-03**
rs16890979	4	9922167	Coding	*SLC2A9*	T	C	−24.654	0.014	**0.042**	0.47	0.087	0.362	0.36	4.80E-03	**0.015**
rs1481012	4	89039082	Intronic	*ABCG2*	G	A	27.555	3.50E-31	**1.02E-29**	1.56	1.27E-10	**3.68E-09**	2.5	5.33E-45	**1.55E-43**
rs2231137	4	89061114	Coding	*ABCG2*	T	C	−16.945	2.33E-12	**3.38E-11**	0.91	0.254	0.56	0.67	8.23E-08	**1.19E-06**
rs231253	4	113362622	3′ UTR	*ALPK1*	C	G	0.121	0.971	0.971	1	1	1	0.99	0.914	0.985
rs742132	6	25607571	Intronic	*LRRC16A*	G	A	−3.605	0.099	0.192	1.1	0.236	0.56	1.02	0.824	0.985
rs9358890	6	25779392	Coding	*SLC17A4*	G	A	−4.741	0.052	0.108	1.27	9.02E-04	**0.013**	1.16	0.023	0.06
rs3799352	6	25822620	Intronic	*SLC17A1*	C	T	−7.456	5.35E-03	**0.017**	0.79	0.015	0.108	0.71	1.57E-04	**1.08E-03**
rs712221	6	152180241	Intronic	*ESR1*	A	T	−5.153	0.028	0.069	1.01	0.888	0.927	0.98	0.796	0.985
rs1178947	7	72850178	3′ UTR	*FZD9*	C	T	−1.042	0.934	0.971	1.04	0.736	0.897	1.02	0.837	0.985
rs1051921	7	73007943	3′ UTR	*MLXIPL*	A	G	−3.973	0.419	0.64	1.05	0.646	0.897	0.98	0.918	0.985
rs4994	8	37823798	Coding	*ADRB3*	G	A	0.728	0.944	0.971	0.82	0.042	0.203	0.84	0.051	0.114
rs2941484	8	76478768	3′ UTR	*HNF4G*	T	C	7.125	3.19E-03	**0.012**	1.09	0.233	0.56	1.28	1.87E-04	**1.08E-03**
rs10821905	10	52646093	5upstream	*A1CF*	A	G	11.401	0.038	0.085	1.47	8.85E-03	0.086	1.61	4.32E-04	**1.57E-03**
rs10749127	10	114849353	Intronic	*TCF7L2*	T	C	−7.631	1.79E-03	**7.42E-03**	1.14	0.11	0.4	0.96	0.593	0.905
rs505802	11	64357072	5upstream	*SLC22A12*	T	C	−1.899	0.482	0.665	0.96	0.681	0.897	0.91	0.233	0.451
rs11602903	11	64358241	5upstream	*SLC22A12*	T	A	−1.517	0.58	0.731	0.98	0.805	0.898	0.93	0.332	0.602
rs11231825	11	64360274	Coding	*SLC22A12*	C	T	−2.035	0.452	0.656	0.97	0.773	0.897	0.91	0.233	0.451
rs12273892	11	64480930	Coding	*NRXN2*	T	A	3.269	0.261	0.46	0.97	0.761	0.897	1.06	0.464	0.747
rs3184504	12	111884608	Coding	*SH2B3*	T	C	5.168	0.943	0.971	0.65	0.77	0.897	0.84	1	1
rs738409	22	44324727	Coding	*PNPLA3*	G	C	−1.595	0.302	0.487	1.03	0.663	0.897	0.98	0.842	0.985

Chr., chromosome. HUA, hyperuricemia. A1, allele 1, effect allele. β values for SNP in serum urate were calculated by linear regression adjusted age and gender. *P* values for SNP in serum urate were calculated by deviance analysis for linear regression adjusted age and gender. *P* values for SNP in hyperuricemia and gout were calculated by Fisher’s exact test in addition model. *P*_FDR_ value for SNP was multiple corrected by FDR method. SNPs were annotated by SNPnexus (http://www.snp-nexus.org/). Because of rs1137070 and rs5953210 in Chromosome X, they cannot be analyzed in this table. When *P*_FDR_ < 0.05, it would be considered as significant and showed in bold.

**Table 2:** Association between genetic variants and serum urate/gout in gender subgroup.

Gender	SNP	Gene	A1	A2	Serum Urate			Gout vs. HUA			Gout vs. Control			Gout vs. H + C		
					β	*P*	*P*_FDR_	OR	*P*	*P*_FDR_	OR	*P*	*P*_FDR_	OR	*P*	*P*_FDR_
**Male**	rs1051921	*MLXIPL*	A	G	−6.276	0.061	0.188	1.01	0.950	1.000	0.90	0.374	0.609	0.93	0.587	0.728
	rs1061622	*TNFRSF1B*	G	T	−2.498	0.416	0.644	1.03	0.773	0.887	1.03	0.723	0.862	1.03	0.700	0.780
	rs10749127	*TCF7L2*	T	C	−5.175	0.045	0.156	1.18	0.057	0.220	1.01	0.877	0.971	1.06	0.405	0.634
	rs10821905	*A1CF*	A	G	10.908	0.071	0.200	1.61	3.41E-03	0.053	1.69	3.98E-04	**2.06E-03**	1.64	4.02E-04	**3.12E-03**
	rs11231825	*SLC22A12*	C	T	−1.858	0.712	0.883	0.97	0.754	0.887	0.93	0.371	0.609	0.94	0.455	0.641
	rs1137070	*MAOA*	C	T	−0.796	0.712	0.883	1.11	0.161	0.367	1.11	0.142	0.294	1.11	0.128	0.296
	rs11602903	*SLC22A12*	T	A	−1.118	0.907	0.965	0.96	0.687	0.852	0.93	0.439	0.648	0.94	0.478	0.645
	rs1178947	*FZD9*	C	T	−2.784	0.332	0.587	1.00	1.000	1.000	0.95	0.699	0.862	0.96	0.789	0.815
	rs12129861	*PDZK1*	A	G	−3.914	0.140	0.333	0.90	0.294	0.536	0.83	0.044	0.124	0.85	0.075	0.194
	rs12273892	*NRXN2*	T	A	2.500	0.567	0.798	1.00	1.000	1.000	1.09	0.376	0.609	1.05	0.523	0.676
	rs1260326	*GCKR*	T	C	9.271	1.11E-04	**8.59E-04**	1.06	0.439	0.680	1.27	7.36E-04	**3.26E-03**	1.19	8.78E-03	**0.034**
	rs1481012	*ABCG2*	G	A	25.859	1.57E-24	**4.87E-23**	1.69	4.99E-12	**1.55E-10**	2.70	9.66E-44	**2.99E-42**	2.27	1.07E-33	**3.32E-32**
	rs16890979	*SLC2A9*	T	C	−14.915	0.084	0.216	0.44	0.042	0.220	0.40	0.020	0.068	0.41	0.018	0.055
	rs2027432	*NLRP3*	A	G	−4.867	0.244	0.474	1.27	0.117	0.367	1.08	0.598	0.843	1.14	0.330	0.602
	rs2070803	*TRIM46*	A	G	6.827	0.039	0.152	1.06	0.498	0.726	1.16	0.075	0.194	1.12	0.142	0.296
	rs2231137	*ABCG2*	T	C	−17.783	1.45E-11	**2.25E-10**	0.87	0.139	0.367	0.63	6.18E-09	**9.58E-08**	0.69	4.26E-06	**6.60E-05**
	rs231253	*ALPK1*	C	G	−1.484	0.353	0.587	1.08	0.389	0.634	1.01	0.938	1.000	1.03	0.704	0.780
	rs2941484	*HNF4G*	T	C	9.156	3.07E-04	**1.90E-03**	1.10	0.253	0.491	1.35	3.26E-05	**2.86E-04**	1.27	1.06E-03	**6.59E-03**
	rs3184504	*SH2B3*	T	C	10.386	0.794	0.890	0.52	0.515	0.726	0.74	1.000	1.000	0.65	0.758	0.810
	rs3799352	*SLC17A1*	C	T	−8.894	3.09E-03	**0.016**	0.79	0.032	0.220	0.67	3.69E-05	**2.86E-04**	0.71	2.90E-04	**2.99E-03**
	rs4971101	*TRIM46*	G	A	6.650	0.030	0.132	1.19	0.049	0.220	1.30	1.35E-03	**5.23E-03**	1.27	3.02E-03	**0.016**
	rs4994	*ADRB3*	G	A	1.839	0.559	0.798	0.81	0.055	0.220	0.85	0.126	0.294	0.84	0.074	0.194
	rs505802	*SLC22A12*	T	C	−1.471	0.804	0.890	0.96	0.655	0.846	0.93	0.393	0.609	0.93	0.455	0.641
	rs5438	*SLC2A5*	T	C	−0.187	0.965	0.965	1.22	0.146	0.367	1.15	0.259	0.501	1.18	0.177	0.343
	rs5953210	*MAOA*	A	G	−0.184	0.965	0.965	1.10	0.239	0.491	1.11	0.139	0.294	1.11	0.143	0.296
	rs712221	*ESR1*	A	T	−3.570	0.185	0.410	1.00	1.000	1.000	1.00	0.972	1.000	1.00	0.973	0.973
	rs738409	*PNPLA3*	G	C	−0.571	0.798	0.890	1.05	0.552	0.744	1.02	0.800	0.918	1.03	0.674	0.780
	rs742132	*LRRC16A*	G	A	−2.715	0.209	0.432	1.14	0.166	0.367	1.03	0.686	0.862	1.06	0.409	0.634
	rs7512998	*NLRP3*	C	T	−1.459	0.594	0.801	1.22	0.164	0.367	1.06	0.650	0.862	1.11	0.407	0.634
	rs780094	*GCKR*	T	C	9.331	8.68E-05	**8.59E-04**	1.08	0.354	0.610	1.28	3.92E-04	**2.06E-03**	1.20	5.35E-03	**0.024**
	rs9358890	*SLC17A4*	G	A	−2.525	0.360	0.587	1.23	6.77E-03	0.070	1.18	0.025	0.078	1.19	0.010	**0.035**
**Female**	rs1051921	*MLXIPL*	A	G	2.018	0.512	0.755	0.96	1.000	1.000	1.01	1.000	1.000	1.00	1.000	1.000
	rs1061622	*TNFRSF1B*	G	T	1.012	0.938	0.949	1.08	0.858	1.000	1.18	0.593	0.973	1.14	0.721	1.000
	rs10749127	*TCF7L2*	T	C	−13.689	8.02E-03	0.062	0.72	0.501	1.000	0.59	0.161	0.776	0.63	0.210	0.887
	rs10821905	*A1CF*	A	G	9.054	0.234	0.604	1.09	0.754	1.000	1.33	0.499	0.973	1.25	0.733	1.000
	rs11231825	*SLC22A12*	C	T	−4.161	0.427	0.661	0.71	0.489	1.000	0.63	0.254	0.876	0.65	0.257	0.887
	rs1137070	*MAOA*	C	T	0.746	0.949	0.949	0.98	1.000	1.000	1.06	0.886	1.000	1.04	0.887	1.000
	rs11602903	*SLC22A12*	T	A	−4.131	0.424	0.661	0.91	0.865	1.000	0.80	0.626	0.973	0.83	0.744	1.000
	rs1178947	*FZD9*	C	T	3.361	0.409	0.661	0.96	1.000	1.000	1.00	1.000	1.000	0.99	1.000	1.000
	rs12129861	*PDZK1*	A	G	−8.623	0.107	0.367	1.19	0.594	1.000	1.18	0.602	0.973	1.18	0.602	1.000
	rs12273892	*NRXN2*	T	A	8.449	0.232	0.604	0.68	0.378	1.000	0.78	0.593	0.973	0.75	0.595	1.000
	rs1260326	*GCKR*	T	C	5.820	0.280	0.661	1.04	1.000	1.000	1.15	0.673	0.994	1.12	0.779	1.000
	rs1481012	*ABCG2*	G	A	30.660	2.41E-11	**7.47E-10**	1.32	0.386	1.000	2.22	6.57E-03	0.180	1.92	0.028	0.432
	rs16890979	*SLC2A9*	T	C	−46.523	7.56E-03	0.062	0.63	1.000	1.000	0.13	1.000	1.000	0.16	1.000	1.000
	rs2027432	*NLRP3*	A	G	5.824	0.943	0.949	0.23	0.167	1.000	0.24	0.175	0.776	0.24	0.178	0.887
	rs2070803	*TRIM46*	A	G	10.988	0.021	0.110	1.45	0.239	1.000	1.92	0.047	0.487	1.75	0.079	0.809
	rs2231137	*ABCG2*	T	C	−11.273	4.71E-03	0.062	0.91	0.868	1.000	0.74	0.434	0.973	0.78	0.529	1.000
	rs231253	*ALPK1*	C	G	5.770	0.186	0.577	0.80	0.634	1.000	0.93	1.000	1.000	0.89	0.875	1.000
	rs2941484	*HNF4G*	T	C	2.492	0.727	0.939	1.59	0.133	1.000	1.64	0.103	0.776	1.61	0.104	0.809
	rs3184504	*SH2B3*	T	C	8.782	0.936	0.949	0.42	1.000	1.000	0.47	1.000	1.000	0.46	1.000	1.000
	rs3799352	*SLC17A1*	C	T	−6.267	0.568	0.800	1.08	0.858	1.000	1.19	0.589	0.973	1.15	0.720	1.000
	rs4971101	*TRIM46*	G	A	10.475	0.046	0.178	1.75	0.065	1.000	2.22	0.012	0.180	2.08	0.015	0.432
	rs4994	*ADRB3*	G	A	−0.013	0.720	0.939	0.52	0.241	1.000	0.49	0.142	0.776	0.50	0.193	0.887
	rs505802	*SLC22A12*	T	C	−4.249	0.384	0.661	0.70	0.394	1.000	0.62	0.254	0.876	0.64	0.257	0.887
	rs5438	*SLC2A5*	T	C	−5.789	0.763	0.939	0.83	1.000	1.000	0.83	1.000	1.000	0.83	1.000	1.000
	rs5953210	*MAOA*	A	G	1.592	0.937	0.949	0.74	0.377	1.000	0.79	0.472	0.973	0.78	0.472	1.000
	rs712221	*ESR1*	A	T	−8.678	0.031	0.135	1.11	0.774	1.000	1.00	1.000	1.000	1.03	1.000	1.000
	rs738409	*PNPLA3*	G	C	−3.785	0.390	0.661	1.35	0.300	1.000	1.25	0.471	0.973	1.28	0.390	1.000
	rs742132	*LRRC16A*	G	A	−3.901	0.421	0.661	1.25	0.505	1.000	1.19	0.628	0.973	1.20	0.521	1.000
	rs7512998	*NLRP3*	C	T	7.200	0.788	0.939	0.65	0.611	1.000	0.72	0.794	1.000	0.70	0.795	1.000
	rs780094	*GCKR*	T	C	4.899	0.391	0.661	1.08	0.885	1.000	1.19	0.574	0.973	1.16	0.674	1.000
	rs9358890	*SLC17A4*	G	A	−8.295	0.015	0.090	1.15	0.665	1.000	0.94	0.889	1.000	1.00	1.000	1.000

A1, allele 1, effect allele. HUA, hyperuricemia. H + C, hyperuricemia plus control. β values for SNP in serum urate were calculated by linear regression adjusted age and gender. *P* values for SNP in serum urate were calculated by deviance analysis for linear regression adjusted age and gender. *P* values for SNP in hyperuricemia and gout were calculated by Fisher’s exact test in addition model. PFDR value for SNP was multiple corrected by FDR method. When *P*_FDR_ < 0.05, it would be considered as significant and showed in bold.

**Table 3:** Association between genetic variants and serum urate in subgroups of BMI and smoking status.

Subgroup	SNP	Gene	A1	A2	Subgroup-1			Subgroup-2			Subgroup-3		
					β	*P*	*P*_FDR_	β	*P*	*P*_FDR_	β	*P*	*P*_FDR_
**BMI**	rs1051921	*MLXIPL*	A	G	−21.273	0.226	0.742	−3.176	0.711	0.764	−2.121	0.654	0.945
	rs1061622	*TNFRSF1B*	G	T	−8.077	0.352	0.742	−6.133	0.104	0.232	0.848	0.712	0.945
	rs10749127	*TCF7L2*	T	C	−9.276	0.792	0.801	−8.278	0.010	**0.029**	−5.490	0.151	0.384
	rs10821905	*A1CF*	A	G	53.677	0.205	0.742	6.270	0.382	0.615	13.138	0.095	0.276
	rs11231825	*SLC22A12*	C	T	17.830	0.518	0.742	−1.577	0.460	0.655	−2.765	0.777	0.945
	rs11602903	*SLC22A12*	T	A	20.188	0.422	0.742	−0.828	0.622	0.751	−2.630	0.783	0.945
	rs1178947	*FZD9*	C	T	−19.382	0.258	0.742	0.629	0.648	0.751	0.349	0.932	0.979
	rs12129861	*PDZK1*	A	G	−4.730	0.607	0.742	−6.398	0.102	0.232	−6.280	0.051	0.165
	rs12273892	*NRXN2*	T	A	36.272	0.036	0.645	0.199	0.959	0.959	4.312	0.349	0.674
	rs1260326	*GCKR*	T	C	−2.982	0.616	0.742	10.384	7.02E-04	**6.78E-03**	6.992	0.021	0.075
	rs1481012	*ABCG2*	G	A	30.979	0.129	0.645	27.344	1.28E-15	**3.71E-14**	26.300	1.86E-14	**5.39E-13**
	rs16890979	*SLC2A9*	T	C	45.018	0.317	0.742	−38.983	3.46E-03	**0.017**	−20.530	0.205	0.458
	rs2027432	*NLRP3*	A	G	−11.539	0.661	0.742	2.878	0.699	0.764	−0.515	0.979	0.979
	rs2070803	*TRIM46*	A	G	−20.087	0.530	0.742	8.033	9.32E-03	**0.029**	10.507	4.09E-03	**0.030**
	rs2231137	*ABCG2*	T	C	−17.470	0.414	0.742	−16.036	2.74E-06	**3.97E-05**	−16.200	5.16E-06	**7.48E-05**
	rs231253	*ALPK1*	C	G	−35.160	0.049	0.645	−1.255	0.749	0.775	2.505	0.757	0.945
	rs2941484	*HNF4G*	T	C	22.048	0.121	0.645	4.576	0.236	0.457	8.990	0.013	0.056
	rs3184504	*SH2B3*	T	C	132.372	0.133	0.645	−7.595	0.614	0.751	14.799	0.761	0.945
	rs3799352	*SLC17A1*	C	T	−24.382	0.096	0.645	−12.323	1.68E-03	**0.010**	−2.600	0.499	0.851
	rs4971101	*TRIM46*	G	A	−23.147	0.382	0.742	8.239	6.95E-03	**0.025**	11.396	1.87E-03	**0.018**
	rs4994	*ADRB3*	G	A	−6.443	0.723	0.777	3.831	0.421	0.642	−1.655	0.622	0.945
	rs505802	*SLC22A12*	T	C	17.830	0.518	0.742	−1.472	0.474	0.655	−2.624	0.814	0.945
	rs5438	*SLC2A5*	T	C	−9.690	0.402	0.742	3.953	0.515	0.679	−5.313	0.159	0.384
	rs712221	*ESR1*	A	T	9.627	0.573	0.742	−3.445	0.365	0.615	−7.648	0.013	0.056
	rs738409	*PNPLA3*	G	C	3.422	0.801	0.801	−3.254	0.103	0.232	−2.183	0.398	0.721
	rs742132	*LRRC16A*	G	A	−14.136	0.344	0.742	−9.919	1.77E-03	**0.010**	0.008	0.961	0.979
	rs7512998	*NLRP3*	C	T	−14.082	0.593	0.742	6.528	0.328	0.595	1.005	0.855	0.954
	rs780094	*GCKR*	T	C	−4.266	0.528	0.742	8.753	5.08E-03	**0.021**	8.456	6.34E-03	**0.037**
	rs9358890	*SLC17A4*	G	A	−4.744	0.665	0.742	−4.400	0.182	0.377	−3.912	0.227	0.470
**Smoke**	rs1051921	*MLXIPL*	A	G	5.971	0.549	0.944	−7.482	0.174	0.463	−4.676	0.896	0.980
	rs1061622	*TNFRSF1B*	G	T	−0.779	0.861	0.944	−6.357	0.111	0.403	1.371	0.766	0.980
	rs10749127	*TCF7L2*	T	C	−2.329	0.665	0.944	−5.628	0.165	0.463	−7.566	0.017	0.072
	rs10821905	*A1CF*	A	G	22.615	0.047	0.227	7.961	0.358	0.606	9.987	0.230	0.477
	rs11231825	*SLC22A12*	C	T	1.368	0.911	0.944	−4.284	0.279	0.539	−0.209	0.878	0.980
	rs11602903	*SLC22A12*	T	A	1.298	0.906	0.944	−3.390	0.390	0.606	0.109	0.936	0.980
	rs1178947	*FZD9*	C	T	11.793	0.211	0.510	−4.968	0.397	0.606	−4.150	0.978	0.980
	rs12129861	*PDZK1*	A	G	5.309	0.676	0.944	−3.177	0.505	0.665	−5.869	0.062	0.157
	rs12273892	*NRXN2*	T	A	6.981	0.130	0.449	−5.118	0.244	0.539	7.146	0.058	0.157
	rs1260326	*GCKR*	T	C	6.767	0.170	0.449	4.493	0.211	0.509	9.632	3.98E-04	**2.88E-03**
	rs1481012	*ABCG2*	G	A	16.925	9.31E-04	**0.027**	15.645	3.97E-05	**5.76E-04**	26.700	6.69E-17	**1.94E-15**
	rs16890979	*SLC2A9*	T	C	10.662	0.850	0.944	−32.004	0.013	0.094	−30.951	0.028	0.091
	rs2027432	*NLRP3*	A	G	4.177	0.247	0.534	−1.521	0.873	0.974	−9.090	0.104	0.231
	rs2070803	*TRIM46*	A	G	18.860	5.09E-03	0.053	0.240	0.916	0.975	7.541	8.22E-03	**0.040**
	rs2231137	*ABCG2*	T	C	−11.602	0.041	0.227	−15.777	2.36E-05	**5.76E-04**	−12.900	4.22E-05	**6.12E-04**
	rs231253	*ALPK1*	C	G	1.271	0.572	0.944	2.679	0.432	0.627	−2.099	0.744	0.980
	rs2941484	*HNF4G*	T	C	1.411	0.865	0.944	11.394	2.08E-03	**0.020**	2.385	0.560	0.855
	rs3184504	*SH2B3*	T	C	28.100	0.633	0.944	−0.371	0.975	0.975	16.018	0.501	0.807
	rs3799352	*SLC17A1*	C	T	−14.807	0.014	0.101	−7.875	0.078	0.403	−3.789	0.480	0.807
	rs4971101	*TRIM46*	G	A	18.853	5.45E-03	0.053	0.930	0.781	0.944	7.705	7.91E-03	**0.040**
	rs4994	*ADRB3*	G	A	1.523	0.855	0.944	2.642	0.661	0.833	5.882	0.271	0.496
	rs505802	*SLC22A12*	T	C	1.746	0.961	0.961	−4.339	0.277	0.539	−0.451	0.778	0.980
	rs5438	*SLC2A5*	T	C	−6.393	0.909	0.944	−4.653	0.463	0.639	−0.205	0.913	0.980
	rs712221	*ESR1*	A	T	−5.993	0.164	0.449	−5.768	0.111	0.403	−6.085	0.065	0.157
	rs738409	*PNPLA3*	G	C	−1.163	0.837	0.944	−3.330	0.365	0.606	2.196	0.780	0.980
	rs742132	*LRRC16A*	G	A	−7.178	0.258	0.534	−6.547	0.095	0.403	−0.708	0.980	0.980
	rs7512998	*NLRP3*	C	T	6.811	0.129	0.449	−1.505	0.838	0.973	−6.488	0.274	0.496
	rs780094	*GCKR*	T	C	6.698	0.156	0.449	4.908	0.175	0.463	9.759	3.85E-04	**2.88E-03**
	rs9358890	*SLC17A4*	G	A	−0.331	0.891	0.944	−0.341	0.945	0.975	−5.700	0.027	0.091

A1, allele 1, effect allele. Subgroup of BMI: 1, Underweight (BMI < 18.5); 2, Normal (18.5 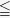 BMI < 25); 3, Overweight (BMI ≧ 25). Subgroup of smoke: 1, non-smokers; 2, former smokers; 3, current smokers. β values for SNP in serum urate were calculated by linear regression adjusted age and gender. *P* values for SNP in serum urate were calculated by deviance analysis for linear regression adjusted age and gender. *P*_FDR_ value for SNP was multiple corrected by FDR method. When *P*_FDR_ < 0.05, it would be considered as significant and showed in bold. Because of rs1137070 and rs5953210 in Chromosome X, they cannot be analyzed in this table.
