# Supplementary material for: Effects of multiple genetic loci on the pathogenesis from serum urate to gout
Source: Sci Rep. 2017 Mar 2;7:43614. doi: 10.1038/srep43614 (PMC5333621; doi:10.1038/srep43614)
Supplement: Supplementary Information [file srep43614-s1.pdf]

Effects of multiple genetic loci on the pathogenesis from serum urate to gout

Zheng Dong[1], Jingru Zhou[1], Shuai Jiang[1], Yuan Li[1], Dongbao Zhao[2], Chengde Yang[3], Yanyun Ma[1], Yi Wang[1], Hongjun He[4], Hengdong Ji[5], Yajun Yang[1,6], Xiaofeng Wang[1,6], Xia Xu[2], Yafei Pang[2], Hejian Zou[7,8], Li Jin[1,6], Jiucun Wang[1,6,8]*

[1] State Key Laboratory of Genetic Engineering, Collaborative Innovation Center for Genetics and Development, School of Life Sciences, Fudan University Jiangwan Campus, Shanghai, China.

[2] Division of Rheumatology and Immunology, Changhai Hospital, Shanghai, China.

[3] Division of Rheumatology, Ruijin Hospital, Shanghai Jiaotong University School of Medicine, Shanghai, China.

[4] Division of Rheumatology, Taixing People's Hospital, Jiangsu Province, China.

[5] Division of Rheumatology, Taizhou People's Hospital, Jiangsu Province, China.

[6] Fudan-Taizhou Institute of Health Sciences, Taizhou, Jiangsu Province, China.

[7] Division of Rheumatology, Huashan Hospital, Fudan University, Shanghai, China.

[8] Institute of Rheumatology, Immunology and Allergy, Fudan University, Shanghai, China.

*Address corresponding to Jiucun Wang, Ph.D, School of Life Sciences, Fudan University, 2005 Songhu Road, Shanghai 200438, People's Republic of China. (e-mail: jcwang@fudan.edu.cn).

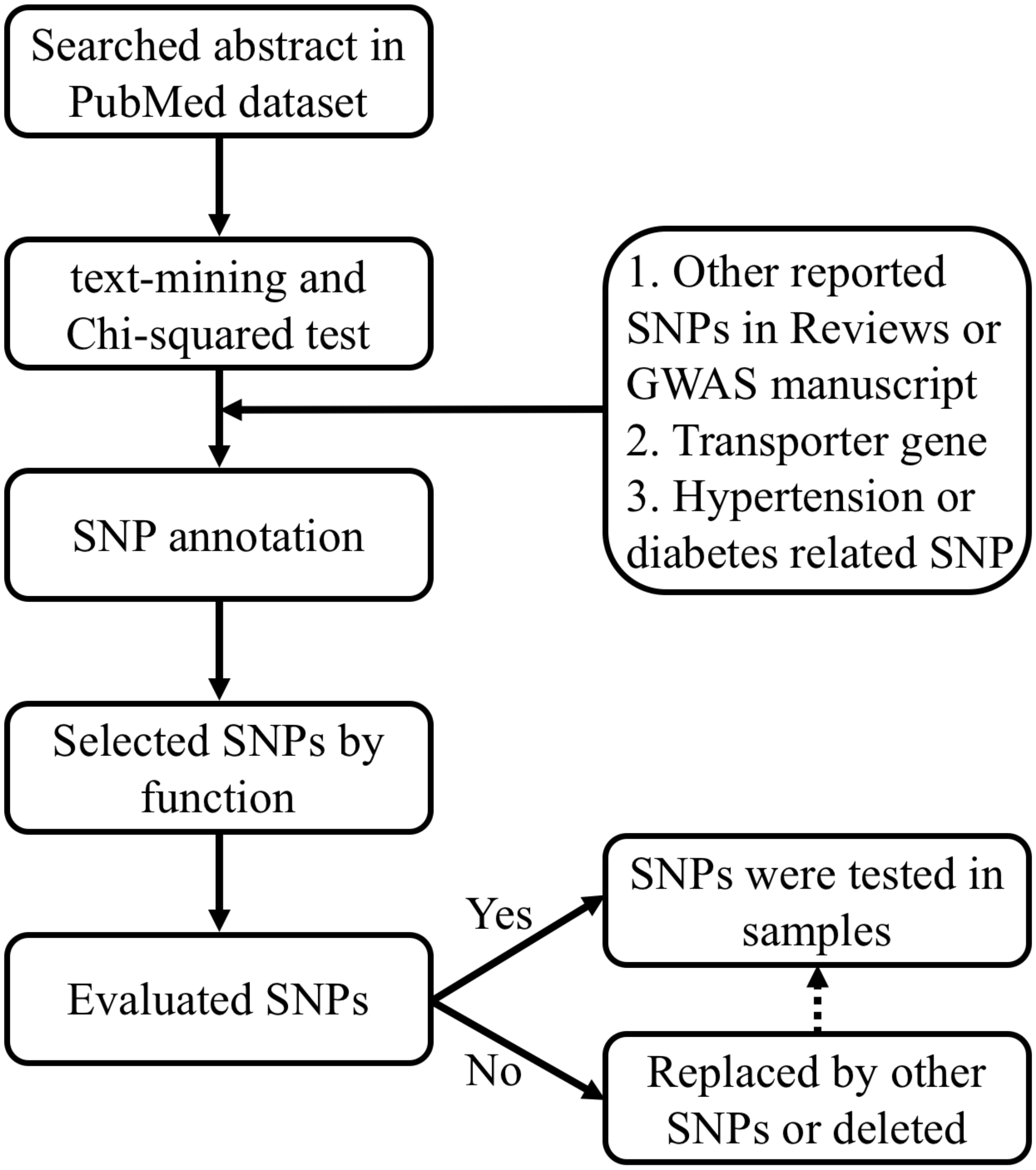

Figure S1. Procedure for selecting target loci. First, SNP association studies were downloaded from the PubMed database (http://www.ncbi.nlm.nih.gov/pubmed/). Second, text-mining technology was used to search for SNPs associated with serum urate levels and/or gout and recorded the frequencies of reported associations for each SNP (frequency 1). Third, the same text-mining method was used to calculate the frequencies of associations with phenotypes other than serum urate levels and gout for each of the above SNPs (frequency 2). Fourth, each of the selected SNPs was considered as a candidate if its frequency 1 was

significantly different with its frequency 2 with a P value less than 0.05 in Chi-squared test. In addition, those candidate SNPs were manually verified. Other reported urate/gout-associated SNPs in published reviews were also enrolled in our study. In addition, other important candidate SNPs in transporter genes and hypertension- or diabetes-related genes were included. All selected SNPs were annotated by SNPnexus (http://www.snp-nexus.org/) and filtered by their SNP functions (i.e. SNPs in 5'-upstream, 5'-utr, coding, intronic, 3'-utr and 3'-downstream were selected). Then, the SNPs were evaluated by the requirements of SNPscan, the genotyping technology used in our study. SNP that did not satisfy the requirements were replaced by other SNP in the same gene or deleted directly. Finally, after filtration, 31 SNPs were treated as target SNPs for further analysis.

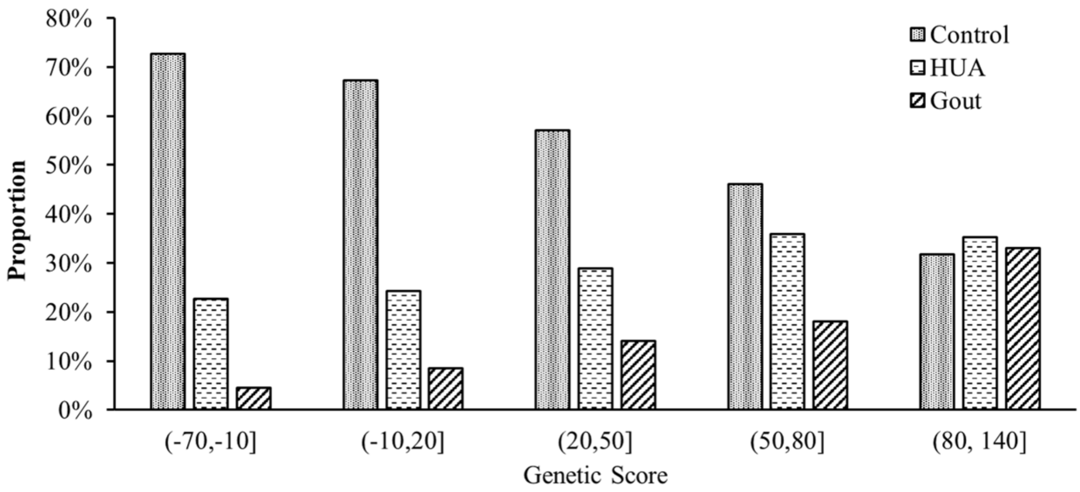

Figure S2. Relationship between genetic urate score and the proportions of hyperuricemia and gout in males.

Table S1. Association for genetic variants and the risk of gouty tophi

SNPs	Genes	*P*	P_{FDR}
rs1051921	*MLXIPL*	0.935	1.000
rs1061622	*TNFRSF1B*	0.168	0.366
rs10749127	*TCF7L2*	0.634	0.876
rs10821905	*A1CF*	4.75E-04	3.44E-03
rs11231825	*SLC22A12*	0.683	0.883
rs11602903	*SLC22A12*	0.750	0.883
rs1178947	*FZD9*	0.761	0.883
rs12129861	*PDZK1*	0.157	0.366
rs12273892	*NRXN2*	0.035	0.129
rs1260326	*GCKR*	6.60E-03	0.038
rs1481012	*ABCG2*	3.23E-21	9.37E-20
rs16890979	*SLC2A9*	0.362	0.525
rs2027432	*NLRP3*	0.899	1.000
rs2070803	*TRIM46*	0.098	0.285
rs2231137	*ABCG2*	1.33E-04	1.93E-03
rs231253	*ALPK1*	0.322	0.492
rs2941484	*HNF4G*	0.062	0.199
rs3184504	*SH2B3*	1.000	1.000
rs3799352	*SLC17A1*	0.013	0.056
rs4971101	*TRIM46*	3.88E-04	3.44E-03
rs4994	*ADRB3*	0.235	0.436
rs505802	*SLC22A12*	0.700	0.883
rs5438	*SLC2A5*	0.240	0.436
rs712221	*ESR1*	0.306	0.492
rs738409	*PNPLA3*	0.177	0.366
rs742132	*LRRC16A*	0.162	0.366
rs7512998	*NLRP3*	0.966	1.000
rs780094	*GCKR*	0.014	0.056
rs9358890	*SLC17A4*	0.260	0.443

P values for loci in gouty tophi were calculated by 3 X 2 count data of genotype. P_{FDR} values for loci were multiple corrected by the FDR method. Because rs1137070 and rs5953210 are located on Chromosome X, they could not be analyzed in this table.

Table S2. Expression quantitative trait loci (eQTL) in two existing expression eQTL databases

**eQTL**	**Gene**	***P* value in database 1**	***P* value in database 2**
rs10821905	*A1CF*	11.658	22.178
rs12129861	*PDZK1*	9.131	5.298
rs1260326	*GCKR*	11.347	11.654
rs2070803	*TRIM46*	19.509	
rs2231137	*ABCG2*		5.236
rs3799352	*SLC17A1*	10.398	8.616
rs4971101	*TRIM46*	19.180	
rs780094	*GCKR*	9.569	11.06
rs742132	*LRRC16A*	9.854	

P value means the max –log10 P-value. Database 1, Genotype-Tissue Expression Data Portal (http://www.gtexportal.org/home/); Database 2, Geuvadis data browser (http://www.ebi.ac.uk/Tools/geuvadis-das/).

Table S3. Characteristics of participants in this study

Characteristics	Control	HUA
Number	2945	1387
Male (%)	70.53%	76.42%
Age	69.00 (9.25)	67.91 (11.93)
Height (cm)	161.37 (7.52)	162.63 (7.54)
Weight (kg)	63.71 (9.96)	68.56 (11.12)
BMI	24.43 (3.24)	25.88 (3.52)
Cigarette smoking (%)	87.05%	72.33%
Serum Urate (umol/l)	300.73 (63.13)	482.30 (61.14)
total bilirubin (umol/l)	19.39 (8.39)	18.98 (7.59)
Glucose (mmol/L)	5.60 (1.67)	5.59 (1.31)
Cholesterol (mmol/L)	4.75 (0.93)	5.06 (1.52)
Triglyceride (mmol/L)	1.51 (0.96)	2.23 (1.76)
Creatinine (umol/L)	71.75 (17.61)	89.80 (29.24)
blood urea nitrogen (mmol/L)	5.48 (2.13)	6.24 (1.95)

HUA, hyperuricemia. Data are shown as the mean (SD). Cigarette smoking included ever smoking and current smoking.

Table S4. Characteristics of participants in this study separated by gender

Characteristics	Control	HUA	Gout
**Male**			
Number	2077	1060	509
Age	70.81 (8.48)	67.25 (12.38)	52.99 (14.51)
BMI	24.31 (3.12)	25.70 (3.39)	25.88 (4.48)
Serum Urate (umol/l)	315.56 (57.26)	483.73 (61.08)	526.26 (104.13)
**Female**			
Number	867	327	52
Age	64.68 (9.58)	70.03 (10.03)	66.50 (17.57)
BMI	24.72 (3.52)	26.46 (3.82)	25.21 (3.92)
Serum Urate (umol/l)	265.36 (58.75)	477.64 (60.99)	453.70 (122.88)
**Total**			
Number	2945	1387	582
Age	69.00 (9.25)	67.91 (11.93)	53.56 (15.12)
BMI	24.43 (3.24)	25.88 (3.52)	25.91 (4.27)
Serum Urate (umol/l)	300.73 (63.13)	482.30 (61.14)	522.67 (107.29)

HUA, hyperuricemia. Data are shown as the mean (SD). Cigarette smoking included ever smoking and current smoking.
